# Privacy-Preserving and Lightweight Selective Aggregation with Fault-Tolerance for Edge Computing-Enhanced IoT

**DOI:** 10.3390/s21165369

**Published:** 2021-08-09

**Authors:** Qiannan Wang, Haibing Mu

**Affiliations:** Key Laboratory of Communication and Information Systems, School of Electronic Information Engineering, Beijing Jiaotong University, Beijing 100044, China; 19120134@bjtu.edu.cn

**Keywords:** Internet of Things (IoT), edge computing, selective aggregation, privacy-preserving, fault tolerance

## Abstract

Edge computing has been introduced to the Internet of Things (IoT) to meet the requirements of IoT applications. At the same time, data aggregation is widely used in data processing to reduce the communication overhead and energy consumption in IoT. Most existing schemes aggregate the overall data without filtering. In addition, aggregation schemes also face huge challenges, such as the privacy of the individual IoT device’s data or the fault-tolerant and lightweight requirements of the schemes. In this paper, we present a privacy-preserving and lightweight selective aggregation scheme with fault tolerance (PLSA-FT) for edge computing-enhanced IoT. In PLSA-FT, selective aggregation can be achieved by constructing Boolean responses and numerical responses according to specific query conditions of the cloud center. Furthermore, we modified the basic Paillier homomorphic encryption to guarantee data privacy and support fault tolerance of IoT devices’ malfunctions. An online/offline signature mechanism is utilized to reduce computation costs. The system characteristic analyses prove that the PLSA-FT scheme achieves confidentiality, privacy preservation, source authentication, integrity verification, fault tolerance, and dynamic membership management. Moreover, performance evaluation results show that PLSA-FT is lightweight with low computation costs and communication overheads.

## 1. Introduction

The rapid development of Internet of Things (IoT) technology has made a considerable impact on our lives, such as smart home [1], smart healthcare [2], and smart grid [3]. More and more IoT devices connect to the Internet, and the cloud center analyzes all sensing data in traditional cloud computing, wherein it is difficult to provide real-time services to meet the requirements of IoT applications [4]. Edge computing is used to preprocess the data at the network edge and then transmit these preprocessed data to the cloud center [5]. Thus, it is introduced into IoT to overcome the bottleneck mentioned above (also regarded as an edge computing-enhanced IoT system) [6]. Owing to the distributed architecture of edge computing, sensitive information can be directly stored and processed on edge devices. Nevertheless, the capacity of the edge device is limited, and edge devices are easily captured by adversaries, resulting in the unreliability of edge devices [7]. Therefore, edge computing may increase the possibility of sensitive information leakage [8].

As an essential data processing technique, data aggregation can reduce energy and bandwidth consumption and gain accurate information by merging redundancy data. Although data aggregation is beneficial to edge computing-enhanced IoT, the adversaries can eavesdrop on messages during the transmission between the entities, and even modify messages and forge signatures. Consequently, the authenticity of aggregated data cannot be guaranteed, and the decision of the cloud center may be disturbed. Therefore, privacy-preserving data aggregation (PPDA) has emerged as a significant research area [9].

Most existing aggregation schemes do not process data before the aggregation to avoid revealing data privacy, i.e., overall aggregation [10,11,12,13,14,15]. However, the overall aggregation will aggregate massive unrelated data, which increases the difficulty of both data analysis and data storage. Aggregating the data selectively within the scope of the query will be more beneficial to reduce response latency. Therefore, many selective aggregation schemes have already been proposed [16,17,18,19,20,21,22]. Nonetheless, both overall aggregation and selective aggregation schemes face the following challenges. Firstly, the accuracy of the aggregated data is likely to have a decrease since some unrelated data are also involved in the data aggregation and influence the final decisions. Secondly, a few schemes do not achieve source authentication and integrity verification, and the messages and signatures may be modified or tempered. Thirdly, the huge computation costs bring challenges to resource-constrained IoT devices. Fourthly, fault tolerance should be taken into account to enhance the availability of the aggregation schemes.

We present PLSA-FT, a privacy-preserving and lightweight selective aggregation scheme with fault tolerance for edge computing-enhanced IoT. Our main contributions are as follows:

- In PLSA-FT, the cloud center can set filtering conditions for the data source to avoid aggregating unrelated data. Hence, selective data aggregation can be achieved by constructing Boolean responses and numerical responses according to the attributes of the data source.
- We have constructed the encryption, the aggregation, and the decryption process on the basis of the modified Paillier homomorphic cryptosystem to ensure the confidentiality and privacy of the individual IoT device’s data.
- The PLSA-FT is fault-tolerant, which means that the cloud center could obtain the aggregated data uploaded by all the working IoT devices, even if some IoT devices fail to upload reports.
- We have analyzed the system characteristics to prove that the PLSA-FT scheme achieves confidentiality, privacy preservation, source authentication, integrity verification, fault tolerance, and dynamic membership management. Furthermore, we have evaluated the performance of the scheme to show that the PLSA-FT is lightweight.

The outline of this paper is as follows. The Section 2 introduces related works. The Section 3 presents the system model, the security model, and design goals. In the Section 4, we describe the proposed PLSA-FT scheme in detail. The Section 5 and the Section 6 demonstrate the system characteristic analyses and the performance evaluation. Finally, we provide a conclusion in the Section 7.

## 2. Related Work

Privacy-preserving data aggregation has attracted much attention in recent years. To protect the sensitive information of users, the homomorphic encryption technology [11,13,16,17,18,19,23,24,25,26,27,28,29,30], the differential privacy technology [17,26,27,31], and the pseudonym technology [12,15,32,33] have mainly been used in aggregation schemes [34].

In [15], Guan et al. utilized pseudonyms and pseudonym certificates to perform secure data aggregation and guaranteed the anonymity of the devices. Nonetheless, the certificate generations and updates were time-consuming. Qian et al. [17] adopted the differential privacy technique to ensure vital privacy preservation and supported selective aggregation to provide online user behavior analysis based on the BGN homomorphic cryptosystem. Mahdikhani et al. [18] employed the Paillier homomorphic encryption to encrypt the reports to avoid the leakage of sensitive information. Moreover, selective aggregation was achieved by computing the inner product similarity to identify the aggregation subset. Zhang et al. [24] constructed a lightweight and verifiable PPDA scheme, called LVPDA, which was proved to be existentially unforgeable under the chosen message attack. LVPDA introduced the edge computing paradigm for efficient data storage and computing services. Nonetheless, the overall interaction of the scheme was complicated, and the signature verification did not support batch verification. In [32], Wang et al. proposed the first anonymous and secure aggregation scheme. In this scheme, the introduction of fog computing transferred storage and computing from the cloud center to fog nodes in order to solve high latency and lack of support for mobility. Moreover, pseudonyms were used for protecting the identities of terminal devices, and homomorphic encryption was employed for guaranteeing data security in fog-based public cloud computing. However, a large number of time-consuming bilinear pairs were used for signature verification, which leads to relatively large computation costs. The security model of this scheme considered that the cloud center was entirely believable, and that the assumption of security needed to be lowered in future work.

However, these schemes mainly focused on privacy, anonymity, and selective aggregation, while the fault tolerance of the scheme was ignored. This could be a large problem because IoT devices are prone to malfunctions. The fault tolerance characteristic was especially significant in [28,29]. Li et al. [28] set the sum of all devices’ secret parameter $\pi_{ij}$ to 0 in order to enhance the security of plaintext $m_{ij}$. Nonetheless, CC would not be able to decrypt the aggregated ciphertexts if one or more IoT devices malfunctioned, since the sum of $\pi_{ij}$ was no longer 0.

Shi et al. [31] proposed a fault-tolerant protocol based on diverse groups. Grining et al. [35] proposed a provable level of privacy even if massive devices malfunctioned. Nonetheless, the above traditional PPDA schemes did not adopt the architecture of edge-computing/fog-computing and suffered from latency problems.

Lu et al. proposed a lightweight PPDA scheme to achieve data aggregation and filter fake data, based on the Paillier homomorphic cryptosystem and the Chinese Remainder Theorem [13]. Even if some devices were malfunctioning, their scheme could support fault tolerance. In [30], Zeng et al. presented a data aggregation scheme, which could support column aggregation and support an additional row aggregation. Furthermore, MMDA was fault-tolerant. However, not all the data was useful, as the aggregation of multi-dimensional data from two directions exacerbated the waste of resources. The schemes mentioned above took advantage of the computational capacity of fog computing/edge computing, whereas selective aggregation was not considered. Selective aggregation was beneficial to the recourse-constrained IoT because it could avoid spending massive resources on the aggregation and storage of unrelated data. However, there is seldom any work aiming to support the fault tolerance for selective data aggregation schemes.

In addition to fault tolerance and selective aggregation, dynamic membership management was also significant for practical application scenarios. In schemes without dynamic membership management, all the entities should be reset when there is any membership updating. It would cost considerable computation and communication overheads. Hence, we proposed the PLSA-FT system to aggregate data according to data source attributes and support the IoT devices’ dynamic membership.

## 3. Models and Design Goals

### 3.1. System Model

**Figure 1:**
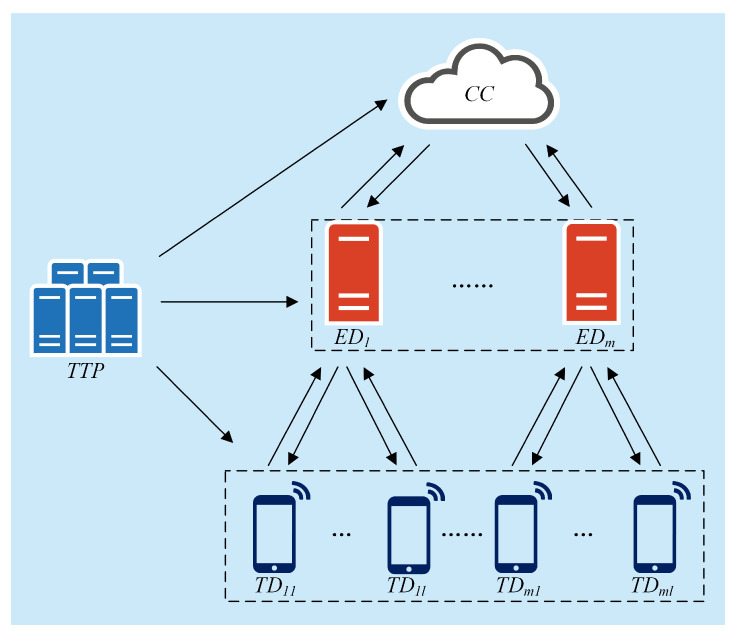
The system model of the proposed PLSA-FT scheme.

In our scheme, we consider a trusted third party, a cloud center, m edge devices, and m×l IoT, which are shown in Figure 1.

The trusted third-party TTP: The TTP is responsible for initialization and assigning keys for all entities in a secure way. If an IoT device participates in or exits the system, the value of the secret parameter θ in the TTP’s database will update. TTP also helps in case of IoT devices’ malfunctioning.

IoT devices $TD=\left\{TD_{11},TD_{12},\cdots ,TD_{m\left(l-1\right)},TD_{ml}\right\}$: $TD_{ij}$ generates responses according to collected data and sends encrypted reports to the corresponding edge device $ED_{i}$.

Edge devices $ED=\left\{ED_{1},ED_{2}\cdots ED_{m}\right\}$: The $ED_{i}$ generally refers to the edge server. Specifically, $ED_{i}$ transmits messages between the cloud center and IoT devices.

Cloud center CC: The CC broadcasts queries to TD s via corresponding edge devices, aggregates ciphertexts from ED s, and analyzes data after decryption.

### 3.2. Security Model

We assume that the trusted third-party TTP is fully trusted, while the cloud center and edge devices are honest but curious, which means that the cloud center and edge devices would try to gain information by analyzing received data without any modification. Each IoT device is considered to be honest in our scheme.

We considered an external adversary who may eavesdrop on the sensitive information during data transmission, initiate reply attacks, and launch active attacks to modify the messages or forge the signatures. Note that PPDA is the focus of this paper. Other active attacks, i.e., denial of service (DoS) attacks and internal adversaries, are beyond the scope of this paper.

### 3.3. Design Goals

The main goal of our scheme is to aggregate data without revealing individual IoT device’s data. At the same time, we hope that the scheme supports fault tolerance and dynamic membership management. Specifically, the design goals can be summarized as follows:

Confidentiality and privacy preservation: Adversaries cannot infer any data from ciphertexts without the decryption key. The cloud center can only recover all IoT devices’ aggregated data, and the individual IoT device’s data are protected.

Source authentication and integrity verification: Every legal entity has a unique identity, and the reports generated from illegal devices could be detected. Meanwhile, if the adversaries modify the data or forge signatures, malicious operations would be detected.

Fault tolerance: Even if one or more IoT devices malfunction, the proposed PLSA-FT scheme can still work as usual.

Dynamic membership management: When new IoT devices join or old ones exit the system, any parameters of other devices need not be updated.

## 4. Our Proposed Scheme

### 4.1. System Initialization

We assume that the TTP will bootstrap the whole system. Given two security parameters $k_{1},k_{2}$, TTP first chooses two random large prime numbers $p_{1},q_{1}$ with $k_{1}-bit$ length and $\left|p_{1}\right|=\left|q_{1}\right|=k_{1}$. Then, let $n=p_{1}\cdot q_{1}$, choose a generator $g_{1}=n+1$ and $g_{1}\in Z_{n^{2}}^{*}.$ Then, define a function L(x)=(x−1)/n, output public key pk=n, and private key sk=λ for encryption and decryption. Then, TTP generates a bilinear map $e:G_{1}\times G_{1}=G_{2}$ of prime order q, where $\left|q\right|=k_{2}$. Then, TTP chooses four secure hash functions $H:{\left\{0,1\right\}}^{*}\to Z_{n}^{*}$, $H_{1}:{\left\{0,1\right\}}^{*}\to G_{1}$, $H_{2}:{\left\{0,1\right\}}^{*}\to Z_{q}^{*}$, $H_{3}:G_{1}\to Z_{q}^{*}$ and a chameleon hash function $H_{CH}:Z_{q\;}^{*}\to G_{1}$. Finally, the TTP publishes the public parameters $\left\{q,e,G_{1},G_{2},H,H_{1},H_{2},H_{3},H_{CH},n,g_{1}\right\}$ to all entities in the system, and keeps sk=λ available to CC.

### 4.2. Registration

The TTP chooses a random number $x\in \;Z_{q\;}^{*}$ as CC’s private key and computes $Y=g^{x}.$ Then, the TTP publishes the public key Y and sends the private key x to CC, and CC keeps its private key secretly. Similarly, TTP selects an identity $ID_{ED_{i}}$ and a random number $x_{i}\in Z_{q}^{*}$ for $ED_{i},$ then computes $Y_{i}=g^{x_{i}}$. Then, TTP  stores $\left\{ID_{ED_{i}},Y_{i}\right\}$ in CC’s database. $ED_{i}$ keeps its private key $x_{i}$ secret. Considering there are m⋅l IoT devices, TTP generates $x_{ij}\in Z_{q}^{*}$,i=1,2⋯m,j=1,2⋯l as $TD_{ij}$’s private key and computes $\theta \in Z_{q}^{*}$ such that

(1) $$\sum_{i=1}^{m}\sum_{j=1}^{l}x_{ij}+\theta \equiv 0\mathrm{mod}\lambda .$$

Only the TTP and IoT devices know the private key $x_{ij}$. The TTP also computes the corresponding $Y_{ij}=g^{x_{ij}}$ for each IoT device in the system and stores $\left\{ID_{TD_{ij}},Y_{ij}\right\}$ in the  CC’s database and in the corresponding $ED_{i}$’s database. When an IoT device joins in the system, it should apply the registration to TTP. When an IoT device exits the system, it should send a message to notify TTP to update the value of secret parameter θ. TTP also regularly inquires of edge devices to obtain the information of working IoT devices to avoid that TTP does not receive the message from the IoT device because of power outages or network fadings.

$TD_{ij}$ further chooses $w_{ij},y_{ij},z_{ij},s_{ij},t_{ij}\in Z_{q\;}^{*}$ and computes

(2) $$r_{ij}=H_{2}\left(ID_{TD_{ij}}∥w_{ij}\right),f=g^{y_{ij}},h=g^{z_{ij}}.$$

$TD_{ij}$ stores the personal information $PI=\left(r_{ij},s_{ij},t_{ij}\right)$, where $s_{ij}$ and $t_{ij}$ are trapdoor keys. Then, $TD_{ij}$ calculates

(3) $$H_{CH_{ij}}=g^{r_{ij}}\cdot f^{s_{ij}}\cdot h^{t_{ij}},$$

and the offline signature

(4) $$sig_{ij}^{off}=H_{1}{\left(H_{CH_{ij}}\right)}^{x_{ij}}.$$

Finally,$\;TD_{ij}$ sends message $\left\{ID_{TD_{ij}},TS_{off},H_{CH_{ij}},sig_{ij}^{off}\right\}$ and verification key (f,g,h) to the corresponding $ED_{i}$, where $TS_{off}$ denotes the current timestamp.

After receiving the message packet from $TD_{ij}$, $ED_{i}$ first checks the validity of $ID_{TD_{ij}}$ and the freshness of $TS_{off}$. Then, $ED_{i}$ performs batch verification $e\left(g,\prod_{j=1}^{l}sig_{ij}^{off}\right)?=\prod_{j=1}^{l}e\left(Y_{ij},H_{1}\left(H_{CH_{ij}}\right)\right)$, significantly reducing the computation costs of $ED_{i}$. If the equation holds, $TD_{ij}$ is valid. Otherwise, $TD_{ij}$ is invalid and $ED_{i}$ rejects $TD_{ij}$’s responses later.

### 4.3. Query Broadcasting

Whenever CC desires, it broadcasts query Q to all IoT devices via intermediate edge devices. The query Q is formally defined as Q=(A∥B), where $A=\left\{a_{1},a_{2},\ldots ,a_{k}\right\}$ contains all query conditions $a_{i}$ of the data source attributes in the current query, B denotes CC’s basic query condition, and ∥ denotes the concatenation function. A query $Q_{1}$ is defined as $Q_{1}=\{A=(female\;\&\;age>60)∥B=heart\;rate\}$, whose query conditions of the data source attribute are “female” and “age > 60”, and the basic query condition is “heart rate”. CC uses its private key x to sign query Q as

(5) $$\sigma =H_{1}{\left(Q∥TS_{q}\right)}^{x}$$

to guarantee that the query Q is not altered, where $TS_{q}$ denotes the current timestamp. Then, CC sends $\left\{Q,TS_{q},\sigma \right\}$ to all IoT devices via corresponding edge devices.

### 4.4. IoT Devices Responses

After each IoT device receives the query, it first checks the freshness of $TS_{q}.$ Then, each IoT device checks the validity of signature σ through the equation $e\left(Y,H_{1}\left(Q∥TS_{q}\right)\right)=e\left(g,\sigma \right)$. The query is accepted when the equation holds. Otherwise, the signature is invalid, and the query is rejected. If the query is accepted, each IoT device $TD_{ij}$ constructs the response $R_{ij}$ on the basis of query Q. Each $TD_{ij}$’s response $\;R_{ij}$ is formally defined as

(6) $$R_{ij}=\left(RB_{ij}∥RN_{ij}\right)$$

$RB_{ij}$ can be computed as $RB_{ij}=$($b_{1}\;\&b_{2}\;\&\cdots \&b_{k})$, and $\;b_{i}$ is the Boolean response to the corresponding query condition of the data source attribute $a_{i}$.$\;RN_{ij}$ denotes numerical response to basic query condition B. Each $TD_{ij}$ runs the Algorithm 1 to obtaian the output $R_{ij}=\left(RB_{ij}∥RN_{ij}\right)$. We define $R=\max\left\{R_{11},R_{12},\cdots ,R_{ml}\right\}$. Note that, the range [0,R] is still a small message space in comparison with $Z_{n}$.
**Algorithm 1: IoT devices responses**  **Input:** $TD_{ij}$’s Boolean response $\left(b_{1},b_{2},\cdots ,b_{k}\right)$ and numerical response $RN_{ij}$  **Output:** $R_{ij}=\left(RB_{ij}∥RN_{ij}\right)$1: **for** each $TD_{ij}$ **do**2:    $RB_{ij}=(b_{1}\&b_{2}\&\cdots \&b_{k})$
3:    **if** $RB_{ij}=1$ **then**4:      $RN_{ij}=RN_{ij}$
5:    **else**6:      $RN_{ij}=0$
7:    **end if**8: **end for**9: **return** $R_{ij}=\left(RB_{ij}∥RN_{ij}\right)$


$TD_{ij}$ computes

(7) $$C_{ij}=E\left(R_{ij}\right)=\left(1+R_{ij}\cdot n\right)\cdot H{\left(TS\right)}^{x_{ij}\cdot n}\mathrm{mod}n^{2},$$

where TS denotes the current timestamp. When $H{\left(TS\right)}^{x_{ij}\cdot n}$ is computed in advance, $TD_{ij}$ only needs to perform multiplication operations. Then, $TD_{ij}$ computes online signature on the basis of $PI=\left(r_{ij},s_{ij},t_{ij}\right)$ as follows:

(8) $$s_{ij}^{*}=\left(\left(r_{ij}-C_{ij}\right)+\left(t_{ij}-t_{ij}^{*}\right)\cdot y_{ij}+s_{ij}\cdot z_{ij}\right)\cdot y_{ij}{}^{-1}.$$

$TD_{ij}$ randomly chooses $t_{ij}^{*}\in \;Z_{q\;}^{*}$, and the online signature $sig_{ij}^{on}=\left(t_{ij}^{*},s_{ij}^{*}\right)$ is formed. Finally, $TD_{ij}$ sends message $\left\{ID_{TD_{ij}},TS,C_{ij},sig_{ij}^{on}\right\}$ to $ED_{i}$.

### 4.5. Edge Device Aggregation

Upon receiving the message from $TD_{ij}$, $ED_{i}$ first checks the timestamp TS and the validity of $ID_{TD_{ij}}$. Then, $ED_{i}$ uses verification key (f,g,h) to check if

(9) $$H_{CH_{ij}}\left(r_{ij},s_{ij},t_{ij}\right)=H_{CH_{ij}}\left(C_{ij},s_{ij}^{*},t_{ij}^{*}\right).$$

The correctness of above equation can be proved as follows:

(10) $$\begin{array}{cc} H_{CH_{ij}}\left(C_{ij},s_{ij}^{*},t_{ij}^{*}\right) & =g^{C_{ij}}\cdot f^{s_{ij}^{*}}\cdot h^{t_{ij}^{*}}\\ & =g^{C_{ij}}\cdot f^{\left(\left(r_{ij}-C_{ij}\right)+\left(t_{ij}-t_{ij}^{*}\right)\cdot y_{ij}+s_{ij}\cdot z_{ij}\right)\cdot y_{ij}{}^{-1}}\cdot h^{t_{ij}^{*}}\\ & =g^{C_{ij}}\cdot g^{y_{ij}\cdot \left(\left(r_{ij}-C_{ij}\right)+\left(t_{ij}-t_{ij}^{*}\right)\cdot y_{ij}+s_{ij}\cdot z_{ij}\right)\cdot y_{ij}{}^{-1}}\cdot g^{z_{ij}\cdot t_{ij}^{*}}\\ & =g^{r_{ij}}\cdot f^{s_{ij}}\cdot h^{t_{ij}}\\ & =H_{CH_{ij}}\left(r_{ij},s_{ij},t_{ij}\right) \end{array}$$

If the equation holds, the message sent by $TD_{ij}$ is valid. Otherwise, the message is invalid. If the message is valid, $ED_{i}$ aggregates the ciphertext by computing

(11) $$C_{i}=\prod_{j=1}^{l}C_{ij}.$$

Then, $ED_{i}$ calculates signature

(12) $$sig_{i}=H_{1}{\left(ID_{ED_{i}}∥TS∥C_{i}\right)}^{x_{i}}.$$

Finally, $ED_{i}$ sends message $\left\{ID_{ED_{i}},TS,C_{i},sig_{i}\right\}$ to CC. Note that if the set $\hat{TD}\subset TD\;$ indicates that the devices in the set do not upload the reports, $ED_{i}$ computes

(13) $$\bar{C_{i}}=\prod_{TD_{ij}\in TD/\hat{TD}\;}C_{ij}\mathrm{mod}n^{2}$$

and the corresponding signature is

(14) $$\bar{sig_{i}}=H_{1}{\left(ID_{ED_{i}}∥TS∥\bar{C_{i}}\right)}^{x_{i}}.$$

Finally, $ED_{i}$ sends message $\left\{ID_{ED_{i}},TS,\bar{C_{i}},\bar{sig_{i}}\right\}$ to CC.

### 4.6. Edge Device Aggregation

After receiving the message packet from $ED_{i}$, CC first checks the validity of $ID_{ED_{i}}$ and the freshness of the timestamps TS. Then, CC performs batch verification $e\left(g,\prod_{i=1}^{m}sig_{i}\right)?=\prod_{i=1}^{m}e\left(Y_{i},H_{1}\left(ID_{ED_{i}}∥TS∥C_{i}\right)\right)$, which significantly reduces the computation costs of CC. If the equation holds, $ED_{i}$ is valid. Otherwise, $ED_{i}$ is invalid and CC checks $e\left(g,sig_{i}\right)?=e\left(Y_{i},H_{1}\left(ID_{ED_{i}}∥TS∥C_{i}\right)\right)$ to identify the invalid message.

If the message is valid, CC sends decryption requirements to TTP, TTP returns $H{\left(TS\right)}^{n\cdot \theta }$ to CC. Then, CC aggregates the ciphertexts by computing

(15) $$\begin{array}{ll} C & =\prod_{i=1}^{m}C_{i}\cdot H{\left(TS\right)}^{n\cdot \theta }\mathrm{mod}n^{2}\\ & =\prod_{i=1}^{m}\prod_{j=1}^{l}\left[\left(1+R_{ij}\cdot n\right)\cdot H{\left(TS\right)}^{x_{ij\cdot n}}\right]\cdot H{\left(TS\right)}^{n\cdot \theta }\mathrm{mod}n^{2}\\ & =\left(1+\sum_{i=1}^{m}\sum_{j=1}^{l}R_{ij}\cdot n\right)\cdot H{\left(TS\right)}^{{}^{\left(\sum_{i=1}^{m}\sum_{j=1}^{l}x_{ij}+\theta \right)\cdot n}}\mathrm{mod}n^{2}\\ & \underset{\to }{\sum_{i=1}^{m}\sum_{j=1}^{l}x_{ij}+\theta \equiv 0\mathrm{mod}\lambda \Rightarrow \sum_{i=1}^{m}\sum_{j=1}^{l}x_{ij}+\theta =\tau \cdot \lambda \;for\;some\;\tau }\\ & =\left(1+\sum_{i=1}^{m}\sum_{j=1}^{l}R_{ij}\cdot n\right)\cdot H{\left(TS\right)}^{\tau \cdot \lambda \cdot n}\mathrm{mod}n^{2}\\ & \underset{⇁}{x^{n\lambda }\equiv 1\mathrm{mod}n^{2}\Rightarrow H{\left(TS\right)}^{\tau \cdot \lambda \cdot n}\equiv 1\mathrm{mod}n^{2}}\\ & =\left(1+\sum_{i=1}^{m}\sum_{j=1}^{l}R_{ij}\cdot n\right)\mathrm{mod}n^{2} \end{array}$$

CC can obtain the aggregated plaintext $\sum_{i=1}^{m}\sum_{j=1}^{l}R_{ij}$ by computing

(16) $$\sum_{i=1}^{m}\sum_{j=1}^{l}R_{ij}=L\left(C\right)=\left(C-1\right)/n.$$

$\sum_{\left\{RB_{ij}=1\right\}}RB_{ij}$ counts the number of IoT devices that satisfy CC’s query conditions. $\sum_{\left\{RN_{ij}\neq 0\right\}}RN_{ij}$ denotes the sum of numerical responses that satisfy CC’s query conditions. CC can further gain the mean $\bar{m}$ of aggregated data by computing

(17) $$\bar{m}=\frac{\sum_{\left\{RN_{ij}\neq 0\right\}}RN_{ij}}{\sum_{\left\{RB_{ij}=1\right\}}RB_{ij}}=\frac{\sum_{i=1}^{m}\sum_{j=1}^{l}RN_{ij}}{\sum_{i=1}^{m}\sum_{j=1}^{l}RB_{ij}}.$$

The correctness of the ciphertext’s aggregation can be proved as follows:

(18) $$\begin{array}{ll} \sum_{i=1}^{m}\sum_{j=1}^{l}R_{ij} & =\overset{m}{\underset{i=1}{\sum }}\sum_{j=1}^{l}(RN_{ij}∥RB_{ij})\\ & =\sum_{\left\{RB_{ij},RN_{ij}=0\right\}}(RB_{ij}∥RN_{ij})+\sum_{\left\{RB_{ij},RN_{ij}\neq 0\right\}}(RB_{ij}∥RN_{ij})\\ & =\sum_{\left\{RB_{ij}=1\right\}}RB_{ij}∥\sum_{\left\{RN_{ij}\neq 0\right\}}RN_{ij} \end{array}$$

### 4.7. Fault Tolerance Handling

If some IoT device $\hat{TD}\subset TD$ cannot work, CC aggregates the ciphertexts as follows:

(19) $$\hat{C}=\prod_{i=1}^{m}\bar{C_{i}}=(\left(1+\sum_{TD_{ij}\in TD\backslash \hat{TD}}R_{ij}\cdot n\right)\cdot H{\left(TS\right)}^{\sum_{TD_{ij}\in TD\backslash \hat{TD}}x_{ij}\cdot n})\mathrm{mod}n^{2}.$$

Even if the equation $H{\left(TS\right)}^{\left(\sum_{TD_{ij}\in TD\backslash \hat{TD}}x_{ij}+\theta \right)\cdot n}\equiv 1$ mod $n^{2}$ does not hold, CC can still use private key λ to obtain aggregated plaintexts $\sum_{TD_{ij}\in TD\backslash \hat{TD}}R_{ij}$. CC computes

(20) $${\hat{C}}^{\lambda }=\left(1+n\cdot \lambda \cdot \sum_{TD_{ij}\in TD\backslash \hat{TD}}R_{ij}\right)\mathrm{mod}n^{2}.$$

The aggregated plaintexts can be recovered by

(21) $$\sum_{TD_{ij}\in TD\backslash \hat{TD}}R_{ij}=L\left({\hat{C}}^{\lambda }\right)=\frac{{\hat{C}}^{\lambda }-1}{n\cdot \lambda }.$$

Similarly, CC can obtain the corresponding mean $\bar{m}$.

### 4.8. Extension to Support Dynamic Membership

Since the IoT devices in the edge computing-enhanced IoT system may change, our scheme can provide dynamic membership management. If some new IoT devices TD∈A participate in the system or some old ones TD∈B exit, TTP will update the value of θ and replace θ with θ′. θ′ can be computed as

(22) $$\theta^{\prime }=\theta -\sum_{TD\in A}x_{ij}+\sum_{TD\in B}x_{ij}\mathrm{mod}\lambda .$$

If some new IoT devices participate in the system, they need to apply the registration to TTP, and the detailed registration operations are described in Section 4.2. If some old IoT devices exit, TTP needs to notify CC and the corresponding $ED_{i}$ to delete the corresponding record $\left\{ID_{TD_{ij}},Y_{ij}\right\}$. The cost of our extension is much less than that of other schemes, which need to update IoT device’s private key.

**Figure 2:**
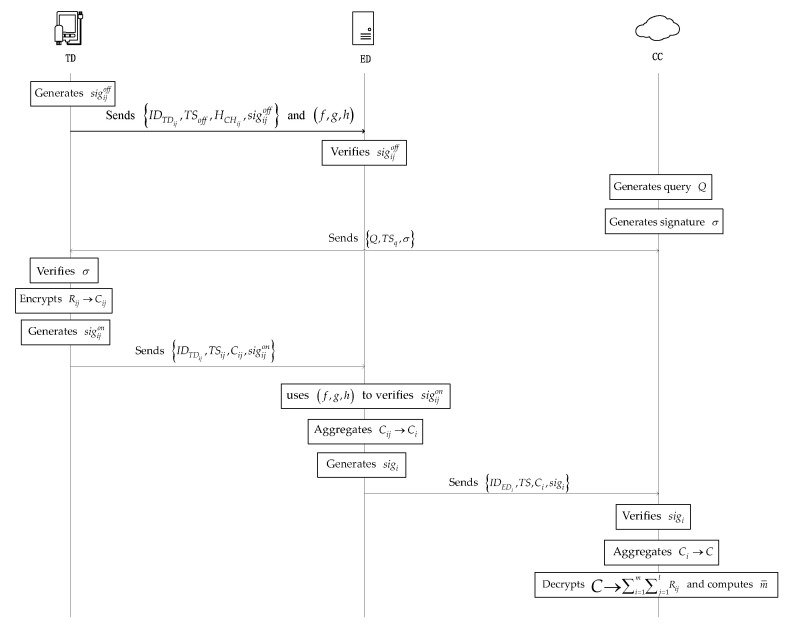
High-level description of the main phase of the PLSA-FT scheme.

The high-level description of the main phase of the PLSA-FT scheme is shown in Figure 2.

We also show the main phases of our proposed PLSA-FA scheme in Table 1.

## 5. System Characteristic Analyses

### 5.1. Confidentiality and Privacy Preservation

**Theorem** **1.** *The privacy of the individual IoT device’s data* $R_{ij}$*cannot be compromised by an external adversary.*

**Proof** **of** **Theorem** **1.** If an external adversary eavesdrops on the communication between $TD_{ij}$ and $ED_{i}$ to obtain the report $C_{ij}$. In PLSA-FT, the $TD_{ij}$ reports its data in the form of $C_{ij}=E\left(R_{ij}\right)=\left(1+R_{ij}\cdot n\right)\cdot H{\left(TS\right)}^{x_{ij}\cdot n}\mathrm{mod}n^{2}$. According to the property under Module $n^{2}$, i.e., ${\left(1+n\right)}^{x}\equiv \left(1+n\cdot x\right)\mathrm{mod}n^{2}$, $C_{ij}$ will become ${\left(1+n\right)}^{R_{ij}}\cdot H{\left(TS\right)}^{x_{ij}\cdot n}\mathrm{mod}n^{2}$. If we let $r=H{\left(TS\right)}^{x_{ij}}$, g=(1+n), and $g\in Z_{n^{2}}^{*}$, then the ciphertext $C_{ij}$ will become $C_{ij}=g^{R_{ij}}\cdot r^{n}\mathrm{mod}n^{2}$ and is still a valid Paillier ciphertext. Since the Paillier encryption algorithm has been proved to be semantically secure against chosen plaintext attacks, an external adversary cannot gain $R_{ij}$ without private key λ. □

**Theorem** **2.** *The privacy of remaining IoT devices is protected, even if a set of IoT devices is comprised.*

**Proof** **of** **Theorem** **2.** If a set of IoT devices are compromised, their corresponding secret keys $x_{ij}$ will be leaked. In PLSA-FT, the TTP randomly generates secret parameters $x_{ij}\in Z_{q}^{*}$, i=1,2⋯m,j=1,2⋯l and there is no correlation between them. In other words, even if an adversary compromises some IoT devices, it has no chance to reveal the secret keys of the remaining IoT devices and the privacy of the remaining IoT devices’ data.

In an extreme case, an adversary successfully compromises m×l−1 IoT devices and obtains their corresponding secret keys $x_{11}$,$x_{12}$,$\cdots x_{ml-1}$(i=1,2⋯m,j=1,2⋯l). Recalling Equation (1), the expression for all IoT devices can be expressed in the form of $\sum_{i=1}^{m}\sum_{j=1}^{l}x_{ij}+\theta \equiv 0\mathrm{mod}\lambda$. If we let $\sum x_{ij}$ denote the obtained secret keys, then the above equation will become $\sum x_{ij}+x_{ml}+\theta \equiv 0\mathrm{mod}\lambda$. This means that only when the adversary obtains the secret parameter θ and the secret key λ of CC will it be able to gain $x_{ml}$. Therefore, we can conclude that, no matter how many IoT devices are compromised, the privacy of other IoT devices is protected. □

**Theorem** **3.** *If the* $ED_{i}$ *is compromised, the privacy of individual IoT device’s data* $\;R_{ij}$ *and aggregated data* $\sum_{j=1}^{l}\;R_{ij}\;$ *is preserved.*

**Proof** **of** **Theorem** **3.** If the $ED_{i}$ is compromised, the adversary can obtain multiple $TD_{ij}$’s ciphertexts $C_{ij}=E\left(R_{ij}\right)=\left(1+R_{ij}\cdot n\right)\cdot H{\left(TS\right)}^{x_{ij}\cdot n}\mathrm{mod}n^{2}$. Similarly, the adversary can obtain the aggregated ciphertext $C_{i}=\left(1+\sum_{j=1}^{l}R_{ij}\cdot n\right)\cdot H{\left(TS\right)}^{\sum_{j=1}^{l}\;x_{ij}\cdot n}\mathrm{mod}n^{2}$. According to the proof of Theorem 1, both the ciphertext $C_{ij}$ and the aggregated ciphertext $C_{i}$ are valid Paillier ciphertexts, which are indistinguishable under chosen plaintext attacks. The $ED_{i}$ does not have the Paillier algorithm’s secret key λ to perform the decryption. Thus, even if the adversary has compromised $ED_{i}$, the privacy of the individual device’s data $R_{ij}$ and the privacy of the aggregated data $\sum_{j=1}^{l}\;R_{ij}$ are both protected. □

**Theorem** **4.** *If* CC *is compromised, the privacy of the individual IoT device’s data* $\;R_{ij}$ *is protected.*

**Proof** **of** **Theorem** **4.** If a strong adversary compromises the CC, it can only reveal the aggregated data. Since CC can only obtain aggregated ciphertexts from ED s, the adversary cannot infer the individual IoT device’s data from the aggregated data. Therefore, even though the adversary compromised the CC, the privacy of the individual IoT device is still preserved. □

### 5.2. Source Authentication and Data Integrity

**Theorem** **5.** *Source authentication and integrity verification of the data are guaranteed in proposed PLSA-FT scheme.*

**Proof** **of** **Theorem** **5.** After $ED_{i}$ receives the message packet $\left\{ID_{TD_{ij}},TS,C_{ij},sig_{ij}^{on}\right\}$ from $TD_{ij}$, $ED_{i}$ first checks the freshness of timestamp TS and the validity of $ID_{TD_{ij}}$. $ED_{i}$ can confirm the message packet generated from which $TD_{ij}$ and further check if the entity is legal. Then, $ED_{i}$ checks if the equation $H_{CH_{ij}}\left(r_{ij},s_{ij},t_{ij}\right)=H_{CH_{ij}}\left(C_{ij},s_{ij}^{*},t_{ij}^{*}\right)$ holds to verify the integrity of data. In our scheme, an online/offline signature is adopted, which has been proved to be is existential unforgeable under chosen message attacks in [24]. Only the adversary with trapdoor keys $\left(y_{ij},z_{ij}\right)$ can easily achieve the collision according to the trapdoor collision property [36]. Thus, an adversary cannot pass $ED_{i}$’s integrity verification without trapdoor keys.

In addition, after CC receives the message packet $\left\{ID_{ED_{i}},TS,C_{i},sig_{i}\right\}$ from $ED_{i}$, CC first checks the freshness of timestamp TS and the validity of $ID_{ED_{i}}$. Therefore, CC can confirm the message packet generated from which $ED_{i}$ and further verify if the entity is legal. This ensures that every packet is from a legal entity and cannot be tampered. CC can performs batch verification $e\left(g,\prod_{i=1}^{m}sig_{i}\right)?=\prod_{i=1}^{m}e\left(Y_{i},H_{1}\left(ID_{ED_{i}}∥TS∥C_{i}\right)\right)$, which greatly reduces the CC’s computation costs. If the above equation does not hold, at least one message reported by $ED_{i}$ is invalid, and CC can check $e\left(g,sig_{i}\right)?=e\left(Y_{i},H_{1}\left(ID_{ED_{i}}∥TS∥C_{i}\right)\right)$ to find invalid messages. If an adversary modifies or forges the data, the above equation would not hold. Thereby, our scheme ensures the source authentication and integrity verification of the data. □

### 5.3. Fault Tolerance

**Theorem** **6.** *Suppose at some time slot, certain IoT devices cannot successfully upload the reports*, CC*can still obtain aggregated data of the rest of normal IoT devices.*

**Proof** **of** **Theorem** **6.** In case certain IoT devices $\hat{TD}$ in subset TD are malfunctioning, these devices cannot successfully upload the reports to the corresponding $ED_{i}$. After aggregating the reports from ED s, the CC can obtain the aggregated report $\tilde{C}$, which only includes the normal IoT devices’ reports. Even if the equation $H{\left(TS\right)}^{\left(\sum_{TD_{ij}\in TD\backslash \hat{TD}}x_{ij}+\theta \right)\cdot n}\equiv 1$ mod $n^{2}$ does not hold, the CC can still perform the decryption to obtain aggregated data by computing $L\left({\hat{C}}^{\lambda }\right)$.

According to the properties under Moduln $n^{2}$, e.g., $\overset{m}{\underset{i=1}{\prod }}\left(1+n\cdot x\right)\equiv \left(1+n\cdot \overset{m}{\underset{i=1}{\sum }}x\right)\mathrm{mod}n^{2}$, ${\left(1+n\cdot x\right)}^{\lambda }\equiv \left(1+n\cdot \lambda x\right)$ mod $n^{2}$ and $x^{n\lambda }\equiv 1\mathrm{mod}n^{2}$, the aggregated ciphertext ${\hat{C}}^{\lambda }$ can be computed as follows:

(23) $$\begin{array}{ll} {\hat{C}}^{\lambda } & =\prod_{TD_{ij}\in TD\backslash \hat{TD}}({\left(1+R_{ij}\cdot n\right)}^{\lambda }\cdot H{\left(TS\right)}^{x_{ij}\cdot n\cdot \lambda })\mathrm{mod}n^{2}\\ & ={\left(1+\sum_{TD_{ij}\in TD\backslash \hat{TD}}R_{ij}\cdot n\right)}^{\lambda }\cdot \prod_{TD_{ij}\in TD\backslash \hat{TD}}H{\left(TS\right)}^{x_{ij}\cdot n\cdot \lambda }\mathrm{mod}n^{2}\\ & =\left(1+\sum_{TD_{ij}\in TD\backslash \hat{TD}}R_{ij}\cdot n\cdot \lambda \right)\cdot \prod_{TD_{ij}\in TD\backslash \hat{TD}}1\mathrm{mod}n^{2}\\ & =\left(1+\sum_{TD_{ij}\in TD\backslash \hat{TD}}R_{ij}\cdot n\cdot \lambda \right)\mathrm{mod}n^{2} \end{array}$$

Hence, CC can compute $L\left({\hat{C}}^{\lambda }\right)=\frac{{\hat{C}}^{\lambda }-1}{n\cdot \lambda }$ to obtain aggregated data $\sum_{TD_{ij}\in TD\backslash \hat{TD}}R_{ij}$.

Therefore, the proposed PLSA-FT scheme is well functioning, even if certain IoT devices malfunction. We can conclude that the PLSA-FT is fault-tolerant. □

### 5.4. Dynamic Membership Management

In PLSA-FT, when a new IoT device $TD_{ij}$ joins in the system, the IoT device applies to TTP. Then, TTP assigns the IoT device a secret key $x_{ij}$ and updates the value of secret parameter θ to $\theta^{\prime }$, which can be computed as $\theta^{\prime }=\theta -x_{ij}\mathrm{mod}\lambda$. When $TD_{ij}$ exits the system, TTP updates the value of secret parameter θ to $\theta^{\prime }$, which can be computed as $\theta^{\prime }=\theta +x_{ij}\mathrm{mod}\lambda$. At the same time, TTP needs to notify the CC and the corresponding $ED_{i}$ to delete the record $\left\{ID_{TD_{ij}},Y_{ij}\right\}$.

It can be seen that the joining or exit of IoT devices does not concern other IoT devices, which requires low computation and communication costs.

## 6. Performance Evaluation

We evaluated the performance of the proposed PLSA-FT scheme in the aspects of the computation costs and the communication overheads. We considered other related aggregation schemes [24,25,30,32] as a comparison. We adopted the Java Pairing Based Cryptography Library (JPBC) to estimate the time costs. We used the Type-A curves as defined in the PBC library for the implementation because the Type-A curves offer the highest efficiency among all types of curves. Table 2 shows the symbol and the meaning of the operations and corresponding time costs. The security parameter q is 160 bits, and the RSA modulus n is set to 1024 bits. In addition, we considered that there are m ED s and each ED corresponds to l TD s. Additionally, the length of timestamp TS and identity ID are all 160 bits. All experiments were implemented on Intel Core i7-4790 CPU @ 2.5 GHz, with 4 GB memory with Ubuntu16.04 operating system.

### 6.1. Computation Costs

**Figure 3:**
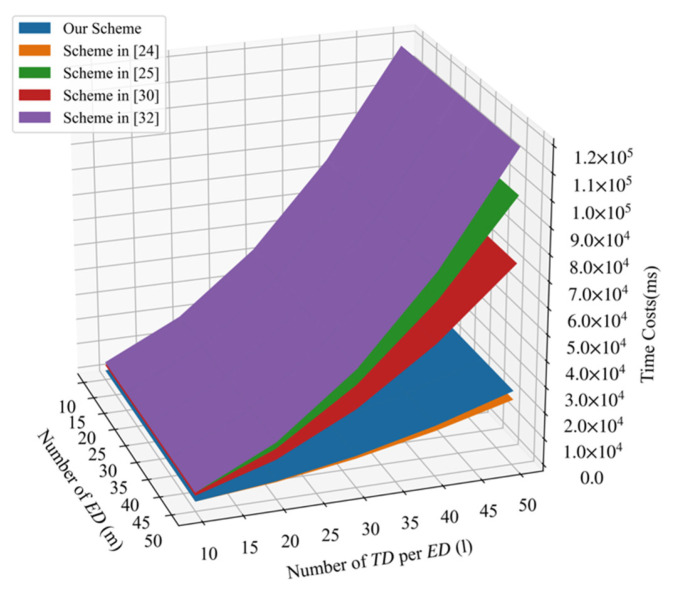
Comparison of overall computation costs.

In PLSA-FT, $\;TD_{ij}$ requires one exponentiation operation in $Z_{n^{2}}$, one hash operation, and three multiplication operations to generate the ciphertext and three multiplication operations in $G_{1}$ to calculate the signature. $ED_{i}$ requires 3l exponentiation operations in $G_{1}$ and 2l multiplication operations to verify the signature $sig_{ij}^{on}$ and l multiplication operations in $G_{1}$ to aggregate ciphertext $C_{i}$, one exponentiation operation, and a hash operation in $G_{1}$ to generate signature $sig_{i}$. CC requires (3m+1) multiplication operations  (m+1) bilinear pairing operations, (m+1) hash operations, and one exponentiation operation in $\;G_{1}$ to verify the signatures and recover the plaintexts. We list a comparative summary of overall computation costs for five schemes in Table 3. From Table 3, we can find that our scheme requires the least $T_{p}$ operations that are the most time-consuming operations. When the number of edge devices increases, the cloud center needs to verify a large number of signatures; thus, the advantage of our scheme will become more evident. Figure 3 shows that the comparison of overall computation costs in terms of the number of TD per ED(l) and the number of ED(m). It shows that our proposed PLSA-FA scheme greatly reduced the overall communication costs. Although the overall computation costs of the scheme [24] are fewer than that of our scheme, our scheme provides more functional properties than that of the scheme [24]. Table 4 further shows the comparison of functionalities achieved by five schemes.

**Figure 4:**
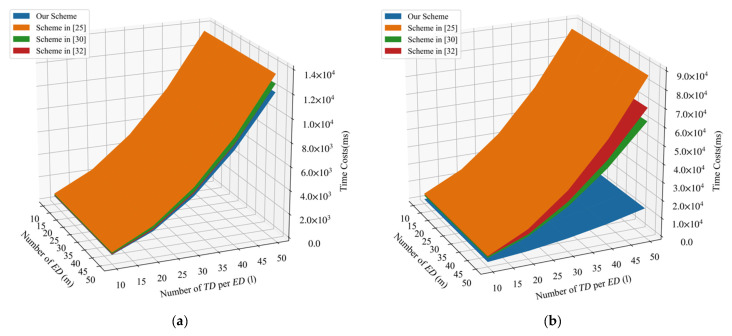
(**a**) Comparison of aggregation costs. (**b**) Comparison of signature and verification costs.

We also compared the computation costs during the aggregation phase in Figure 4a. It can be seen that our scheme requires the least computation costs during aggregation phrase. Figure 4b further depicts the signature and verification costs in terms of the number of TD per ED(l) and the number of ED(m). The time costs of the signature and verification in our proposed PLSA-FA scheme were found to be the least among the four schemes discussed.

### 6.2. Communication Overheads

The communication process of PLSA-FT consists of two processes. One is the communication process from $TD_{ij}$ to $ED_{i}$, and the other one is the communication process from $ED_{i}$ to CC. In the phase of IoT devices responses, each $TD_{ij}$ sent a message packet $\;\left\{ID_{TD_{ij}},TS,C_{ij},sig_{ij}^{on}\right\}$ to $ED_{i}$, and the corresponding communication overheads were 160 + 160 + 2048 + 160 = 2528 bits. Moreover, in the phase of edge device aggregation, each $\;ED_{i}$ sent message packet $\;\left\{ID_{ED_{i}},TS,C_{i},sig_{i}\right\}$ to CC, and the corresponding communication overheads were 160 + 160 + 2048 + 160 = 2528 bits. Considering that there were m edge devices and each $ED_{i}$ corresponded to l IoT devices, the total communication overheads in the scheme were 2528ml+ 2528m bits.

**Figure 5:**
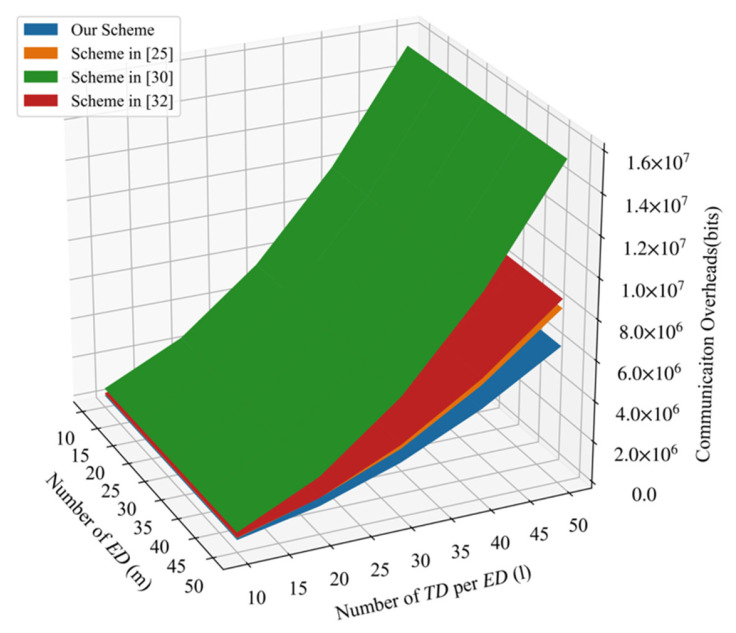
Comparison of communication overheads.

Figure 5 shows the comparison of total communication overheads among four schemes. We can conclude that the PLSA-FT scheme requires the least communication overheads.

## 7. Conclusions

In this paper, we present a privacy-preserving and lightweight selective aggregation scheme with fault tolerance (PLSA-FT) for edge computing-enhanced IoT. PLSA-FT can filter data according to data source attribute to achieve selective aggregation and provide fault tolerance and dynamic membership management. Moreover, benefiting from edge computing, PLSA-FT transfers time-consuming operations to edge devices while reducing the online computatiDon costs. Detailed system characteristic analyses illustrate that the proposed PLSA-FT scheme is secure. Moreover, performance analysis results showed that it is lightweight in both computation costs and communication overheads. However, PLSA-FT is vulnerable to the collusion attacks of edge devices and malicious IoT devices, which exposes the data privacy of a single IoT device. In our future work, we plan to extend our scheme to cope with collusion attacks. Moreover, we also prepare to improve the security properties under more powerful adversaries and active attack models.

## Figures and Tables

**Table 1:** The proposed PLSA-FA scheme.

Registration	$TD_{ij}$ $\mathrm{Generates}\;w_{ij},y_{ij},z_{ij},s_{ij},t_{ij}\in Z_{n\;}^{*}$
	Computes $r_{ij}=H_{2}\left(ID_{TD_{ij}}∥w_{ij}\right)$,$f=g^{y_{ij}}$, $h=g^{z_{ij}}$ Sends $\left\{ID_{TD_{ij}},TS_{off},H_{CH_{ij}},sig_{ij}^{off}\right\}$ and verification key (f,g,h) to $ED_{i}$
	$ED_{i}$ Performs the batch verification $e\left(g,\prod_{j=1}^{l}sig_{ij}^{off}\right)?=\prod_{j=1}^{l}e\left(Y_{ij},H_{1}\left(H_{CH_{ij}}\right)\right)$
	If the equation holds, $TD_{ij}$ is valid. Otherwise, $ED_{i}$ will reject $TD_{ij}$’s reports later
CC	Generates the query Q=(A∥B) and the signature $\sigma =H_{1}{\left(Q∥TS_{q}\right)}^{x}$
Query	Sends $\left\{Q,TS_{q},\sigma \right\}$ to all IoT devices via corresponding edge devices
$TD_{ij}$	Performs the verification $e\left(Y,H_{1}\left(Q∥TS_{q}\right)\right)?=e\left(g,\sigma \right)$
Encryption	If the equation holds, $TD_{ij}$ constructs the response according to Algorithm 1.
	Computes $C_{ij}=E\left(R_{ij}\right)=\left(1+R_{ij}\cdot n\right)\cdot H{\left(TS\right)}^{x_{ij}\cdot n}$ and generates a random number $t_{ij}^{*}\in \;Z_{q\;}^{*}$
	Computes $s_{ij}^{*}=\left(\left(r_{ij}-C_{ij}\right)+\left(t_{ij}-t_{ij}^{*}\right)\cdot y_{ij}+s_{ij}\cdot z_{ij}\right)\cdot y_{ij}{}^{-1}$ and $sig_{ij}^{on}=\left(t_{ij}^{*},s_{ij}^{*}\right)$
	Sends $\left\{ID_{TD_{ij}},TS_{ij},C_{ij},sig_{ij}^{on}\right\}$ to $ED_{i}$
$ED_{i}$	Performs the batch verification $H_{CH_{ij}}\left(r_{ij},s_{ij},t_{ij}\right)?=H_{CH_{ij}}\left(C_{ij},s_{ij}^{*},t_{ij}^{*}\right)$
Aggregation	Aggregates the reports $C_{i}=\prod_{j=1}^{l}C_{ij}$ and generates the signature $sig_{i}=H_{1}{\left(ID_{ED_{i}}∥TS_{i}∥C_{i}\right)}^{x_{i}}$
	Sends $\left\{ID_{ED_{i}},TS_{i},C_{i},sig_{i}\right\}$ to CC
CC	Performs the batch verification $e\left(g,\prod_{i=1}^{m}sig_{i}\right)?=\prod_{i=1}^{m}e\left(Y_{i},H_{1}\left(ID_{ED_{i}}∥TS_{i}∥C_{i}\right)\right)$
Decryption	Sends the decryption requirements to TTP to get $H{\left(TS\right)}^{n\cdot \theta }$
	Aggregates the reports $C=\prod_{i=1}^{m}C_{i}\cdot H{\left(TS\right)}^{n\cdot \theta }$
	Recover the aggregated plaintexts $\sum_{i=1}^{m}\sum_{j=1}^{l}R_{ij}=L\left(C\right)=\left(C-1\right)/n$
	Computes the mean value $\bar{m}=\sum_{i=1}^{m}\sum_{j=1}^{l}RN_{ij}/\sum_{i=1}^{m}\sum_{j=1}^{l}RB_{ij}$
Fault tolerance	If some IoT devices $\hat{TD}\subset TD$ do not work, $ED_{i}$ aggregates the reports
	$\bar{C_{i}}=\prod_{TD_{ij}\in TD/\hat{TD}}C_{ij}$ and generates the signature $\bar{sig_{i}}=H_{1}{\left(ID_{ED_{i}}∥TS_{i}∥\bar{C_{i}}\right)}^{x_{i}}$
	CC aggregates the reports $\hat{C}=\prod_{i=1}^{m}\bar{C_{i}}=\left(1+\sum_{TD_{ij}\in TD\backslash \hat{TD}}R_{ij}\cdot n\right)\cdot \prod_{TD_{ij}\in TD/\hat{TD}}H{\left(TS\right)}^{x_{ij\cdot n}}$ and
	recovers the aggregated plaintexts $\sum_{TD_{ij}\in TD\backslash \hat{TD}}R_{ij}=L\left({\hat{C}}^{\lambda }\right)=\frac{{\hat{C}}^{\lambda }-1}{n\cdot \lambda }$

**Table 2:** Time costs of the operations.

Symbol	Meaning	Time (ms)
$T_{e_{1}}$	Exponentiation in $Z_{n^{2}}$	1.58
$T_{e_{2}}$	Exponentiation in $G_{1}$	1.62
$T_{m}$	Multiplication in $G_{1}$	0.06
$T_{p}$	Bilinear pairing in $G_{1}$	17.62
$T_{h}$	Hash in $G_{1}$	2.97

**Table 3:** The overall computation costs comparison.

Scheme	Overall Computation Costs
Our scheme	$\left(m+1\right)T_{p}+\left(ml+2m+1\right)T_{h}+mlT_{e_{1}}+\left(9ml+3m+1\right)T_{m}+\left(3ml+m+1\right)T_{e_{2}}$
Scheme in [24]	$\left(ml+2m+1\right)T_{p}+\left(2ml+2m\right)T_{h}+\left(5ml+3m\right)T_{m}+\left(4ml+2m+1\right)T_{e_{2}}$
Scheme in [25]	$\left(7ml+2m\right)T_{e_{2}}+\left(7ml+3m\right)T_{m}+\left(ml+4m+2\right)T_{p}+\left(3ml+3m\right)T_{h}$
Scheme in [30]	$\left(ml\right)T_{e_{2}}+\left(6ml+3m\right)T_{m}+\left(2m\right)T_{e_{1}}+\left(3ml+4m\right)T_{h}+\left(ml+3m\right)T_{p}$
Scheme in [32]	$\left(3ml+m\right)T_{e_{2}}+\left(4ml+3m\right)T_{m}+\left(2ml+2m\right)T_{p}+\left(2ml+m\right)T_{h}$

**Table 4:** Comparison of overall computation costs.

Functionality	Our Scheme	Scheme in [24]	Scheme in [25]	Scheme in [30]	Scheme in [32]
Privacy	✓	✓	✓	✓	✓
Integrity verification	✓	✓	✓	✓	✓
Authentication	✓	✓	✓	✓	✓
Fault tolerance	✓	✕	✕	✓	✕
Selective aggregation	✓	✕	✕	✕	✕
Dynamic membership	✓	✕	✕	✕	✕

## Data Availability

Not applicable.
